# Rearing experience with ramps improves specific learning and behaviour and welfare on a commercial laying farm

**DOI:** 10.1038/s41598-021-88347-9

**Published:** 2021-04-23

**Authors:** Kate I. Norman, Claire A. Weeks, John F. Tarlton, Christine J. Nicol

**Affiliations:** 1 Bristol Veterinary School, University of Bristol, Langford House, Bristol, BS40 5DU UK; 2 Royal Veterinary College, Hawkshead Lane, Hatfield, AL9 7TA Hertfordshire UK

**Keywords:** Zoology, Animal behaviour, Ecology, Behavioural ecology

## Abstract

To access resources in commercial laying houses hens must move between levels with agility to avoid injury. This study considered whether providing ramps during rear improved the ability of birds to transition between levels. Twelve commercial flocks (2000 birds/flock) on a multi-age site were examined between 1 and 40 weeks of age. All birds had access to elevated perching structures from 4 days of age. Six treatment flocks were also provided with ramps during rear to facilitate access to these structures. Flocks were visited three times during rear and three times at lay to record transitioning behaviour and use of the elevated structures, together with scores for keel bone and feather damage. Ramp reared flocks used the elevated structures to a greater extent at rear (P = 0.001) and at lay, when all flocks had ramps, showed less hesitancy [i.e. pacing (P = 0.002), crouching (P = 0.001) and wing-flapping (P = 0.001)] in accessing levels. Mean levels of keel bone damage were reduced in ramp reared flocks (52%) compared with control flocks (64.8%) at 40 weeks of age (P = 0.028). The early life experience of the ramp reared flocks enabled specific learning that translated and persisted in later life and resulted in overall welfare benefits.

## Introduction

Cage-free commercial laying hens are housed in complex systems, where resources such as food, water, nest boxes, perches and litter are commonly distributed on different levels. Additionally, in free-range systems, access to the outdoor range can sometimes mean moving through elevated pop-holes. Resources such as litter and the outside range are required for the performance of foraging^1^ and dust bathing; which, together with night-time perching have been identified as behavioural needs^2^. Restriction from these resources can reduce welfare and lead to the development of problems such as feather pecking, fearfulness and aggression^3–5^. It is thus important that hens can move freely throughout such cage-free systems so that each resource is accessible and that welfare benefits are maximised.

In commercial systems evidence suggests that movement around the system is compromised, with reduced ranging observed in birds that roost furthest from pop-holes^6^ and observations of collisions and falls in multi-tier systems^7^. The resultant keel bone injuries restrict movement^8^ and are painful, as shown when Nasr et al.^9^ provided analgesics to birds with keel bone fractures and found that movement from perches was partially restored. Between 39 and 87% of laying hens have evidence of past keel bone fractures depending on house design^10^.

Some studies have looked at provision of ramps between levels in the laying house to facilitate movement and reduce injuries. Pettersson et al.^11^ observed more behaviours indicative of hesitancy such as crouching, pacing and stepping on the spot when birds attempted to move between levels with no ramp provision compared to areas with ramps. Alongside these benefits, the installation of ramps and platforms in commercial aviary systems has been found to reduce collisions by 59%, falls by 45% and keel bone fractures by 23% at 60 weeks of age^12^. As providing birds with access to ramps facilitates movements and reduces the number of fractures during the laying period, it is important to consider how to maximise these benefits and to explore factors that could further increase ramp use.

The importance of rearing in an environment similar to the laying system is increasingly recognised^13^. It is now common practice to provide elevated structures during rearing to promote the development of the skills and behaviours required for pullets to navigate a three-dimensional system in the laying house. There has been limited research about the best type of elevated structures to provide for younger birds. In commercial rearing systems often relatively simple structures such as raised perches are included, but the age that, and extent to which chicks will access these structures has not been researched in a commercial setting.

Given the growing pool of research investigating the benefits of providing ramps for laying hens, we have been studying the effects of ramp provision for younger birds too. In a small-scale study, we found that chicks reared with ramp access from 3-weeks-old in a commercial farm utilised ramps within a small experimental facility to a greater extent and with more confidence when tested at 14 weeks of age than birds reared without ramps^14^. This study also found that layer pullets preferred grid ramps over ladder ramps and showed fewer hesitancy behaviours on grids^15^. One reason why ramps may not routinely be provided to young chicks in commercial systems is that producers want chicks to stay near the heat source, food and water, or because they believe chicks will not use elevated structures during the first few days of life. However, we have shown that chicks will use ramps by day 1, then low-level perches (10 cm) at approximately 5 days of age, and will slowly progress to higher levels (60 cm) by 8 days of age^16^. Facilitated early access to elevated structures has cognitive benefits for young birds^16^.

These studies are promising but have not yet been replicated on a commercial scale. Indeed, replicated longitudinal studies on commercial rearing and laying farms are still rare, and in a commercial setting, it can often prove difficult to use controlled experimental designs. In previous controlled experimental studies of other factors such as stocking density, it has not been feasible to recruit more than six flocks per replicate^17^. By working with a commercial partner, we were able to design a long-term study, using matched flocks of laying hens and this degree of flock-level replication. One aim of this study was to assess the extent to which knowledge and results obtained from experimental studies would apply within commercial systems. Our specific objectives were to examine (i) the effects of ramp provision at rear on the use of elevated structures by young pullets, (ii) the effects of ramp provision at rear on the ability and confidence to use ramps provided to all birds during the laying period and (iii) the effects of ramp provision at rear on welfare parameters including plumage condition, keel bone fractures, floor eggs and production measures during the late rearing and laying periods.

## Results

### Behavioural assessments—elevated structure use at rear

The total number of chicks observed using the elevated structures was significantly different between the treatment groups (Z = 3.621, n = 192, p = 0.001) and the three recording ages (Z = 2.617, n = 192, p = 0.009) with no interaction effects. A greater mean (± SD) number of chicks were observed on the structures in the ramp reared (RR) groups (21.50 ± 17.44) compared to the control reared (CR) groups (13.99 ± 14.25) at all ages. Post hoc test revealed an increase in total structure use between 1 and 3 weeks of age and 1 and 16 weeks of age. But there was no difference in structure use between 3 and 16 weeks of age (See Table 1). Although there appear to be some differences between the rearing treatments at the three ages, an interaction effect was not found, which may be due to the sample size of 12.

**Table 1:** The raw mean number of chicks observed on the 6 elevated structures (ES) (totalled for each area of the ES excluding the number of chicks on the ramps) for the RR group, CR group and the total mean structure use between groups to allow age comparisons.

Age (weeks)	Ramp reared (mean ± SD)	Control reared (mean ± SD)	Total mean ES use (mean ± SD)
1	2.38 ± 5.85	0.00 ± 0.00	1.19 ± 4.27
3	31.84 ± 13.55	17.76 ± 13.91	24.81 ± 15.36
16	28.20 ± 13.55	23.43 ± 10.95	25.82 ± 12.45
Total	21.50 ± 17.44	13.99 ± 14.26	17.74 ± 16.33

Considering the components comprising the elevated structures separately (perches and slatted platforms), significantly greater mean (± SD) number of RR chicks (9.58 ± 8.62) were observed on the slatted platforms compared to the CR chicks (3.62 ± 4.50; z = 4.249, n = 192, p = 0.001). There were no effects of age on the number of chicks using the slats. There were no significant differences between the rearing groups of the number of chicks observed on the perches. However, there was a difference between the three age points (Z = 3.436, n = 192, p = 0.001). Post hoc tests revealed a significant increase in the mean (± SD) number of chicks using the perches with age: 0.008 ± 0.065 at 1 week, 13.52 ± 9.07 at 3 weeks and 18.19 ± 8.84 at 16 weeks.

### Behavioural assessments—movement up or down structures at rear

Age and treatment both affected the route taken to move up and down the structures. For movement up the structures there was a significant difference between treatments with more CR chicks (mean ± SD) using the low perch (5.306 ± 3.82 vs 2.33 ± 2.44; Z = 3.534, n = 72, p = 0.001), and regardless of treatment a greater overall use of the low perch at 3 weeks compared to 16 weeks of age (4.77 ± 4.01 vs 2.86 vs 2.66; Z = − 2.279, n = 72, p = 0.023). More CR chicks were also observed using the low slat to move up the structures (1.00 ± 1.43 vs 0.44 ± 0.97; Z = 1.961, n = 72, p = 0.050). There was no difference between age or treatment for use of the middle perch, high perch, middle slat or high slat to access the structures. Although no comparison between treatments could be made for ramp use, as a reference the mean number of chicks moving up the low-level ramp at 3 weeks of age was 6.22 ± 7.27 and 2.39 ± 3.55 at 16 weeks of age. The mean number using the middle ramp at 3 weeks of age was 6.63 ± 7.49 and 4.56 ± 4.68 at 16 weeks of age.

For movement down the structures more CR chicks (mean ± SD) were observed using the low perch (3.47 ± 2.37 vs 2.39 ± 2.63; Z = 1.951, n = 72, p = 0.051) and regardless of treatment more chicks transitioned down using the low perch at 3 weeks of age (4.08 ± 2.85 vs 1.78 ± 1.51; Z = − 4.152, n = 72, p = 0.001) compared to 16 weeks of age. There were no interaction effects for age and treatment. No age or treatment effects were seen for movements down from the middle perch, high perch, low slat, middle slat and high slat. For reference, the number of RR chicks using the low ramp for access down was 2.94 ± 3.59 at 3 weeks of age and 0.94 ± 1.63 at 16 weeks of age. The mean number using the middle ramp at 3 weeks of age was 4.94 ± 5.61 and at 16 weeks of age 3.22 ± 3.34 for the middle ramp.

### Behavioural assessment—focal bird behaviours at rear

Table 2 illustrates the statistically significant results for age and treatments for movements up and down the elevated structure at rear. When recording focal movement down the elevated structures a greater percentage of crouching and wing flaps were observed at 3 weeks of age compared to 16 weeks of age, but jumps down from slats were greater at 16 weeks of age compared to 3 weeks of age. Jumps down from slats and perches were greater in the CR groups compared to the RR groups.

**Table 2:** Results for focal behaviour transitions. Separated for downwards and upwards transitions. Analysis was conducted for control reared (CR) and ramp reared (RR) treatments at the ages of 3 and 15–16 weeks of age.

**Downwards**	
Crouching	Age: 54.8% (3 weeks) vs 21.2% (16 weeks), Z = − 3.444, n = 695, p = 0.001
Wing flaps	Age: 82.2% (3 weeks) vs 24.0% (16 weeks), Z = − 2.838, n = 695, p = 0.005
Perch jumps	Treatment: 82.7% (CR) vs 42.1% (RR), Z = 9.348, n = 695, p = 0.001
Slat jumps	Treatment: 17.3% (CR) vs 7.5% (RR), Z = 3.206, n = 695, p = 0.001 Age: 15.5% (16 weeks) vs 8.8% (3 weeks), Z = 2.222, n = 695, p = 0.026
**Upwards**	
Pacing	Age: 3.5% (3 weeks) vs 0.3% (16 weeks), Z = − 2.464, n = 704, p = 0.014
Crouching	Treatment: 34.3% (CR) vs 13.3% (RR), Z = 1.860, n = 704, p = 0.063 Age: 39.0% (3 weeks) vs 8.8% (16 weeks), Z = − 3.181, n = 704, p = 0.001
Wing flaps	Treatment: 39.8% (CR) vs 17.7% (RR), Z = 2.583, n = 704, p = 0.010 Age: 43.9% (3 weeks) vs 13.8% (16 weeks), Z = − 2.567, n = 704, p = 0.010
Perch jumps	Treatment: 83.1% (CR) vs 32.2% (RR), Z = 6.820, n = 704, p = 0.001
Slat jumps	Age: 18.3% (16 weeks) vs 5.5% (3 weeks), Z = 2.753, n = 704, p = 0.006

For focal movements up the structures there was a greater percentage of pacing, crouching and wing flaps at 3 weeks of age compared to 16 weeks of age, but the percentage of jumps up to slats was greater at 16 weeks of age compared to 3 weeks. The CR crouched and wing flapped more than the RR birds when transitioning up a structure and performed more perch jumps up the structures.

### Behavioural assessments—level transitions at lay

There was no treatment effect on the number of birds transitioning up or down between the slatted area and the litter. Generally, the mean (± SD) number of hens transitioned more at 24 weeks of age (0.63 ± 0.67) compared to 17 weeks of age (0.24 ± 0.28; Z = 3.193, n = 96, p = 0.001). More birds were also observed transitioning up at 24 weeks of age (0.49 ± 0.47) than at 17 weeks of age (0.22 ± 0.22; Z = 2.329, n = 96, p = 0.020) but there were no age differences for transitioning down.

**Figure 1:**
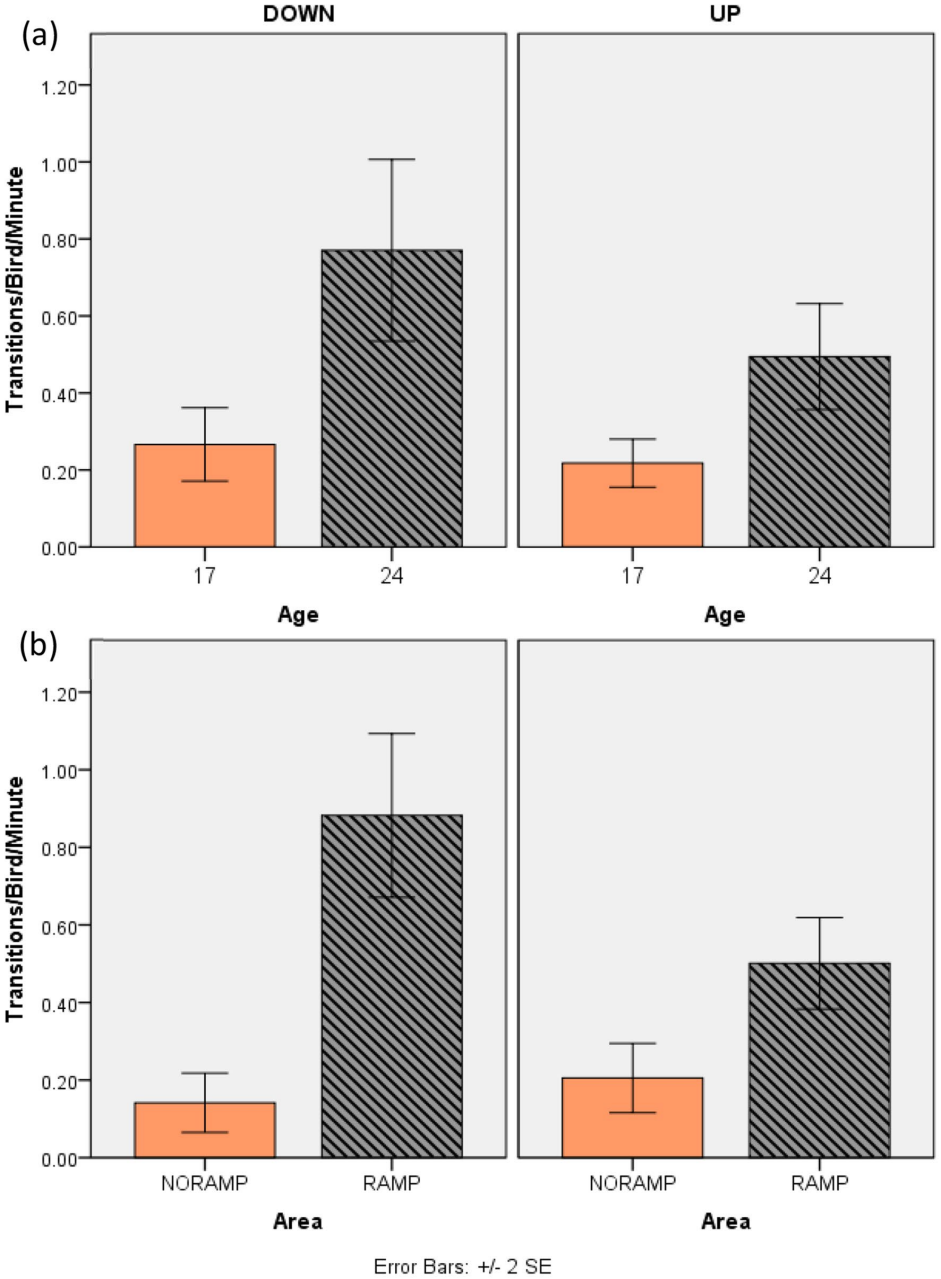
Transitions per bird per minute for **(a)** up and down transitions at 17 and 24 weeks of age and **(b)** up and down transition in ramp and no ramp areas.

Interestingly we found significant differences between areas with ramps and those without in the number of birds that transitioned per minute (adjusted for the number of birds in the recording area) between the slats and litter. More birds (mean ± SD) transitioned down using a ramp area (RA) (0.88 ± 0.73) compared to a no ramp area (NRA) (0.14 ± 0.26; Z = 8.741, n = 96, p = 0.001). There was also a greater number of birds transitioning up in the RA (0.50 ± 0.41) versus NRA (0.21 ± 0.31; Z = 5.399, n = 96, p = 0.001). See Fig. 1 for results.

### Behavioural assessments—focal bird recordings at lay

**Figure 2:**
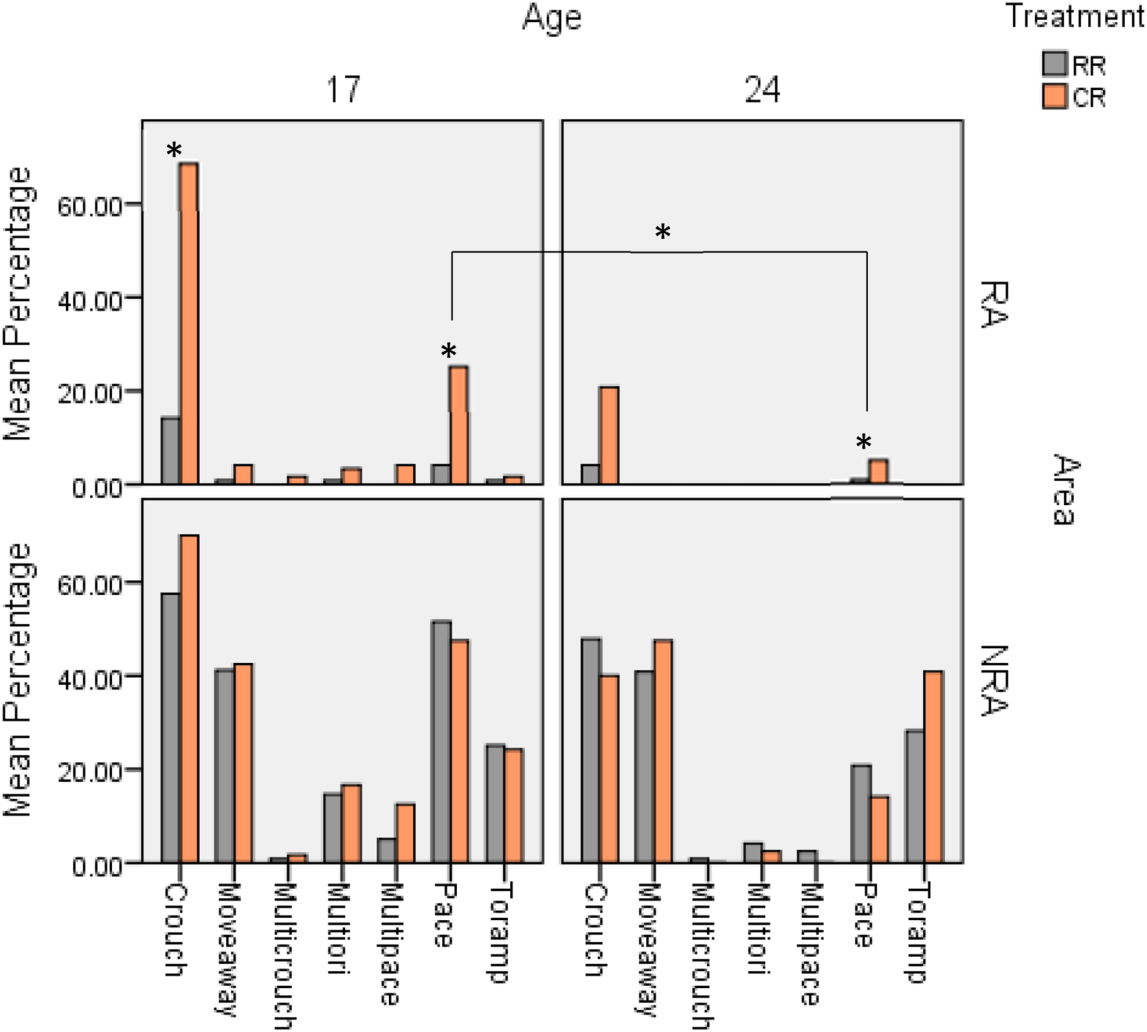
Graphs showing the percentage of focal behaviours for downwards transitions allowing comparisons between age (17 and 24 weeks of age), area (ramp area = RA or no ramp area = NRA) and treatment (ramp reared = RR and control reared = CR). Significant differences between the rearing treatments and age are indicated by an asterisk (*).

For focal behaviour recordings at lay, we first looked for differences in transitions between a RA and NRA. If a difference was found, we continued analysis separately for these areas. Table 3 illustrates the statistically significant results for upwards and downwards transitions. Figure 2 illustrates these differences graphically comparing the ramp areas, age and treatment groups.

**Table 3:** Results for focal behaviour transitions. Separated for downwards and upwards transitions.

**Downwards**	
**Pre transition**	
Pacing	Area: 33.5% (NRA) vs 8.8% (RA), Z = 9.153, n = 957, p = 0.001 Treatment in RA: 15% (CR) vs 2.5% (RR), Z = 3.058, n = 480, p = 0.002 Age in RA: 31.9% (17 weeks) vs 10.3% (24 weeks), Z = − 6.088, n = 408, p = 0.001
Crouching	Area: 54.1% (NRA) vs 26.9% (RA), Z = 8.402, n = 957, p = 0.001 Interaction treatment*age in RA: At 17 weeks 69.2% (CR) vs 14% (RR), Z = 7.131, n = 480, p = 0.001
Multiple pacing	Area: 5% (NRA) vs 1% (RA), Z = 3.242, n = 957, p = 0.001 Age 17 weeks: 8.8% (NRA) vs 1.3% (RA); Z = − 2.486, n = 479, p = 0.013 No other significant differences
**Transition behaviours**	
Wing flaps	Treatment in RA: 1.7% (RR) vs 19.2% (CR), Z = 4.621, n = 467, p = 0.001
Straight down	Treatment in RA: 88.7% (RR) vs 83.0% (CR), Z = − 1.893, n = 467, p = 0.058 Age in RA: 78.9% (17 weeks) vs 92.5% (24 weeks), Z = 2.668, n = 467, p = 0.008
Zig zag	Age in RA: 22% (17 weeks) vs 7.9% (24 weeks), Z = − 2.563, n = 467, p = 0.010
**Move away behaviours**	
Move away	Area: 42.8% (NRA) vs 1.3% (RA), Z = 9.650, n = 210, p = 0.001
Pacing	Age: 43.4% (17 weeks) vs 12.5% (24 weeks), Z = − 3.739, n = 210, p = 0.001
Crouching	Age: 50.0% (17 weeks) vs 5.8% (24 weeks), Z = − 3.178, n = 210, p = 0.001
**Upwards**	
**Pre transition**	
Pacing	Area: 14.5% (NRA) vs 0.83% (RA), Z = 5.748, n = 927, p = 0.001 No significant treatment or age effects
Crouching	Area: 58.6% (NRA) vs 2.7% (RA), Z = 12.913, n = 927, p = 0.001 No significant treatment or age effects
**Transitions**	
Wing flaps	Treatment in RA: 2.1% (RR) vs 8.4% (CR), Z = 3.025, n = 477, p = 0.002 Age in RA: 9.2% (17 weeks) vs 1.3% (24 weeks), Z = − 2.808, n = 477, p = 0.005
**Move away behaviours**	
Move away	Area: 15.7% (NRA) vs 0.2% (RA), Z = 4.515, n = 71, p = 0.001
To ramp	Age: 88.5% (24 weeks) vs 35.6% (17 weeks), Z = 2.632, n = 71, p = 0.008
Multi-head orientations	Age: 28.9% (17 weeks) vs 3.8% (24 weeks), Z = -2.294, n = 71, p = 0.022

Transition area (no ramp area = NRA and ramp area = RA) were analysed first, if a difference was found analysis was continued separately for both treatment (control reared = CR and ramp reared = RR) and age (17 weeks and 24 weeks). Only statistically significant results are presented.

For downwards pre-transitions, we observed more pacing at the NRA compared to the RA. In the RA there was significantly more pacing in the CR compared to the RR birds. The percentage of pacing observed also reduced from 17 weeks of age to 24 weeks of age. A greater percentage of crouching before transitions was observed in the NRA compared to the RA. If an interaction between age and treatment was found, further analysis looked at the simple effects of age and treatment. More crouching was observed in the RA at 17 weeks of age in the CR compared to the RR birds, however, no treatment differences were observed at 24 weeks of age or in the NRA. Overall, more birds were recorded performing multiple pacing behaviour in the NRA compared to RA with large differences at 17 weeks of age in NRA but not at 24 weeks. No differences were found for any other recorded behaviours.

For transition behaviour moving down in a RA, more CR birds wing flapped compared to the RR birds. The number of birds that transitioned in a straight line down the ramp appeared to be approaching significance, more RR birds compared to the CR birds went straight down a ramp. For comparing the difference over age more birds were observed to zig zag down a ramp at 17 weeks of age compared to 24 weeks of age and more birds moved straight down the ramp at 24 weeks of age compared to 17 weeks of age.

Overall, more birds indicated a transition and then moved away without transitioning down in a NRA compared to a RA. For birds that moved away from transitioning more pacing was recorded at 17 weeks compared to 24 weeks. Similarly, more crouching was recorded at 17 weeks of age compared to 24 weeks. For upwards pre-transition behaviours more pacing was observed in the NRA compared to the RA and more crouching in the NRA, however, there were no treatment or age effects. For behaviours observed when transitioning up a RA the RR birds showed fewer wing flaps compared to the CR birds and more wing flaps were observed at 17 weeks of age compared to 24 weeks of age. There were no differences in transitions at the NRA or for any other behaviours recorded. For transitions up more birds moved away in a NRA compared to a RA. More 24-week-old birds moved away to a ramp to transition up. More multiple head orientations were observed at 17 weeks of age compared to 24 weeks of age.

### Welfare and production measures at rear and lay

We observed no treatment effects for feather cover or feather pecking when measured at 16–17 and 24 weeks of age. For the fearfulness tests, we did not observe any treatment differences in the novel object test for the time to approach (Z = − 0.879, n = 96, P = 0.379) or the number of birds interacting with the novel object after the 60 s (Z = 1.156, n = 96, P = 0.248) and no differences in approach distance (Z = − 1.184, n = 240, P = 0.236). There were no differences in the number of floor eggs between the rearing treatments (t_(10)_ = − 0.392, P = 0.759). There were no differences in feather cover scores between the rearing treatments at 40 weeks of age (Z = − 0.470, n = 800, P = 0.638), however, a difference between treatments in birds recorded with keel bone fractures at 40 weeks of age was observed, (Z = − 2.193, n = 800, p = 0.028) with 64.8% of birds recorded with fractures in the CR compared to 52% in the RR groups.

We observed some differences in the welfare measures over the three recording ages. Feather cover was influenced by age (Z = 5.183, n = 640, p = 0.001), post hoc test revealed poorer feather cover at 24 weeks compared to 16 weeks of age and 17 weeks of age. For feather pecking there was a significant difference between the ages and the total pecks observed per bird per second (Z = − 1.966, n = 126, p = 0.049). Post hoc tests revealed an increase in the total number of pecks between 16 and 17 weeks of age and a reduction in pecks between 17 and 24 weeks. There was also a difference between ages in the number of gentle feather pecks (GFP) per bird per second (Z = − 3.007, n = 126, p = 0.003). Post hoc tests show a reduction in GFP between 16 and 24 weeks of age and a reduction between 17 and 24 weeks of age. For the approach test, we observed a shorter response distance of 10.21 cm ± 12.87 (± SD) at 24 weeks of age compared to 16.27 cm ± 15.93 (± SD) at 17 weeks of age (Z = − 2.326, n = 240, p = 0.020).

No difference was found between the rearing treatments for body weight and cumulative percentage mortality. As expected, there was an increase in body weight as the birds aged from 3 to 8 weeks and 14 weeks. Cumulative mortality did not differ between the CR and the RR flocks at the end of rear. At lay there was no differences between the treatment groups in percentage of eggs laid daily, average egg weight, body weight or feed conversion ratio. There was a difference between the ages 20, 30 and 70 weeks in all production parameters as expected (see Table 4).

**Table 4:** Production data statistical analysis for control reared (CR) and ramp reared (RR) flocks at rear (3, 8, 14 weeks of age) and at lay (20, 30 and 70 weeks of age), raw means ± SD.

Rear	Treatment (CR and RR)	Age (3, 8, 14 weeks)
Body weight (grams)	F_(1,32)_ = 0.648, P = 0.427 CR = 754.06 ± 430.52 RR = 760.5 ± 429.55	F_(2,32)_ = 5435.48, P < 0.001 3 weeks = 258 ± 19.06 8 weeks = 734.42 ± 26.89 14 weeks = 1279.42 ± 25.01
Cumulative % mortality	t_(10)_ = -0.194, P = 0.850 CR = 3.39% RR = 3.66%	N/A

Lay	Treatment (CR and RR)	Age (20, 30, 70 weeks)
Eggs (% house daily)	F_(1,31)_ = 0.095, P = 0.760 CR = 58.75% RR = 57.02%	F_(2,31)_ = 298.110, P < 0.001 20 weeks = 11.79% 30 weeks = 89.97% 70 weeks = 73.25%
Average egg weight (grams)	F_(1,22)_ = 0.589, P = 0.451 CR = 64.46 ± 5.08 RR = 64.51 ± 5.51	F_(2,22)_ = 16.927, P < 0.001 20 weeks = 55.16 ± 10.92 30 weeks = 63.64 ± 1.40 70 weeks = 67.95 ± 1.11
Body weight (grams)	F(_1,26)_ = 0.053, P = 0.819 CR = 1612.4 ± 205.89 RR = 1583.2 ± 199.06	F_(2,26)_ = 204.37, P < 0.001 20 weeks = 1612.67 ± 44.17 30 weeks = 1895.14 ± 60.76
Feed conversion ratio	F_(1,22)_ = 0.165, P = 0.688 CR = 8.06 ± 17.35 RR = 16.71 ± 38.87	F_(2,22)_ = 31.20, P < 0.001 20 weeks = 82.16 ± 53.41 30 weeks = 3.91 ± 0.67 70 weeks = 2.61 ± 0.19

## Discussion

The results from this study show that providing ramps during rear in a commercial facility enables chicks to access elevated structures during their first week of life, confirming previous experimental results^14^. Chicks had a preference to use the ramps to access elevated structures and this was observable throughout the rearing period. When transferred to the laying shed a preference for ramps to transition between levels was shown regardless of the rearing treatment. However, there were also long-term effects of early experience with ramps, including a lower percentage of hesitancy behaviours (pacing and crouching before transitioning and wing flaps during a transition) in birds with prior ramp experience suggesting that these birds were more confident in transitioning down ramps in the laying shed. The reduction in hesitancy behaviours was specifically apparent when birds transitioned down in ramp areas at 17 weeks of age, suggesting learning is associated with a specific task (i.e. transitioning using a ramp) rather than being a generalised improved navigational ability resulting from earlier access to elevated structures. There was a significant reduction in keel bone damage for the ramp reared group at 40 weeks of age compared to the control group.

Our findings demonstrate that if ramps are provided during rearing chicks will use elevated structures more. These findings correspond with previous experimental work where we observed chicks from 1-day old using structures with low-level perches (10 cm) and access ramps and observed increased ramp use to 3 days of age followed by an increase in the use of higher structural elements up to 26 days of age^16^. Evidence from these two studies show that chicks can use low perches or ramps from a young age with no consequential practical problems for the farmer. There were no differences at rear in mortality or weight between the ramp reared and control reared birds, suggesting no difficulties in accessing feed, water, or heaters in the commercial rearing system. Similarly, at lay there were no differences in percentage of egg production, egg weight, floor eggs, body weight or feed conversion ratio suggesting no effects of these rearing experiences on production parameters in a single tier system.

Chicks reared without ramps showed a trend towards more crouching before transitioning up and more wing-flapping behaviour than those reared with ramps. We also observed more low perch jumps up the structures and more jumps down from the low-level perches and slats in the control groups. Behaviours indicating hesitancy have been identified and used in both commercial and experimental studies to look at ramp transitions^11,14,15^. In this study, the difference in hesitancy like behaviours between the rearing groups suggests that providing access ramps improves the confidence to transition up and down structures. We found that regardless of treatment the recordings of hesitancy behaviours (crouching, wing flaps, pacing) reduced from 3 to 16 weeks of age when moving up the structures and the occurrence of crouching and wing flaps reduced for movements down the structures.

Encouraging the use of ramps during the rearing period may also have benefits in bone and muscle development. Dial^18^ discussed wing assisted incline running (WAIR) observed in wild Galliformes during movements up inclines. Dial and Jackson^19^ also suggest that adult Galliformes may prefer WAIR rather than jumping or flying up to elevated areas. WAIR has been recorded in domestic laying hens when transitioning inclined ramps of 40 degrees^20^. This wing flapping and running up inclined structures may improve muscle and bone development^21^. Early access to ramps may also improve cognition and spatial navigation^16^ thus improved utilisation of the three-dimensional laying environment.

When transferred to the laying environment differences between the rearing groups were identified in the percentage of hesitancy behaviours observed when transitioning down the ramps. These behaviours have been identified and used in both commercial and experimental studies to look at ramp transitions^11,14,15^. The control group showed more crouching, pacing and wing flapping behaviours before transitioning down a ramp, indicating greater hesitancy. In our previous experimental study, we found birds with ramp experience showed fewer hesitancy behaviours before transitioning (fewer head orientations), during a transition (fewer pauses, turns and returns) and were faster to complete a transition^14^. We have now demonstrated that similar results are found on a commercial farm, and this appears to be particularly important during the first weeks after the transition to the laying environment (17 weeks of age). Smooth transitions into the laying shed and minimising environmental differences have been found to have welfare benefits and reduce stressors at lay^13,22^. It is important to note that the recordings on-farm were taken after 1 week of acclimatisation to the laying shed, this shows a strong effect of rearing with ramps. By 24 weeks of age, we saw significantly more straight down transitions regardless of rearing treatment suggesting improved movement on ramps with experience in both groups. Hesitancy or difficulty in transitioning the ramps (as seen in the control group) could result in restricted access to resources, which are provided at different levels and therefore unsatisfied behavioural needs which could result in frustration and welfare consequences^2^. Although not quite significant, a greater percentage of ramp reared birds moved straight down the ramp compared to the controls, which tended to zig zag at 17 weeks and wing-flapped significantly more. This suggests smoother transitions and more controlled movements down the ramps.

We observed 42.8% of birds moved away from transitioning down levels without ramps compared to 1.3% birds in areas with ramps. Similarly, for upward transitions, 15.7% of birds moved away from areas without ramps compared to 0.2% in areas with ramps. This shows an overall preference for using ramps to transition both up and down between the litter and raised slatted area. No treatment differences were observed between transitions in the no ramp area, suggesting that access to elevated structures at rear did not improve general navigation but improved specific learning for transitioning using a ramp. It appears birds prefer to walk up and down an inclined surface rather than to jump or fly. With an increase in the number of multi-tier laying systems this is an important observation. If birds have trouble in transitioning between levels, they may not access some resources. By simply adding ramps into the system we may improve movement throughout and increasingly commercial multi-tier systems are being designed with ramps. There is evidence that birds with keel fractures are less willing to jump down from a perch for food^8^. Installing ramps in multilevel systems may be important to aid movement of birds with fractures.

An important finding of this study was a significant difference in the percentage of birds with keel bone fractures between rearing groups. In the CR groups 64.8% of the birds were recorded with keel bone fractures and 52% in the RR groups. There is research that suggests rearing in complex environments helps to develop bone mineral density^23,24^ and spatial navigation^16,25^. Our study provided early access to structures requiring spatial navigation and therefore more movement during the first weeks of life. Further work is required to disentangle these theories. Previous research has found that access to ramps in the laying period reduces the number of keel bone fractures^12,26^, thus by providing ramps during rear and lay we may reduce the overall percentage of birds sustaining keel bone fractures. It is important to replicate this work in systems such as multi-tier with high reported percentages of fractures^10,27^.

Restriction from resources such as litter and the outside range have been shown to increase the risk of feather pecking developing within a commercial flock^28^ and it will be observed earlier with more dramatic changes in the environment^29^. We found no difference in feather pecking and feather cover when measured at 17, 24 and 40 weeks of age. We had hypothesised that a difference in feather cover may be observed due to improved access to the litter and range upon transfer to the laying shed, with improved ease and experience of transitioning using ramps. Early access to perches has been suggested to reduce fearfulness in laying flocks^5^. Further, birds reared in more complex environments, such as aviaries, show reduced fearfulness^30^. With the difference in feather cover we might expect to see a difference in the outcome of the fearfulness tests in the respective rearing treatments: as this can be a predictor to feather pecking^31,32^, however we did not see any differences in the approach distance, the response to a novel object or feather cover. This may be due to the simplicity of the system with only one transition level for birds to access the litter area and range. The effects of early rearing experience may be even more important for birds housed in multi-level systems.

In conclusion, we found where ramps are provided at rear chicks will use raised structures from an earlier age and show fewer hesitancy behaviours when transitioning at lay. These results suggest that chicks will learn to utilise specific aspects of their environment from a young age, concluding the importance of rearing in environments that match the laying set up. An overall preference to use a ramp for upwards and downwards transitions suggests that by increasing the number of ramps in the rearing and laying system we may encourage more movement between the litter and raised slatted areas, especially important for multi-tier systems where birds must travel further and make more transitions to access the litter (and range). Increased transitions at early lay may result in improved welfare, as indicated by reduced keel bone fractures in birds provided ramps during rear. Overall, our previous experimental results appear to have translated well into commercial systems giving confidence in the benefits of rearing with ramps in commercial systems.

## Methods

### Experimental design

Over 3 years, six paired organic British Blacktail flocks with intact beaks (i.e. not beak-trimmed) were visited between 1 and 40 weeks of age. Within each pair, one flock was ramp reared (RR) and one flock was control reared without ramps (CR). All flocks were kept on one farm which possessed two rearing houses and six laying sheds of approximately 2000 birds per flock. The site was multi-age, meaning that of the six laying sheds there were three different ages on the site at one time.

The availability of this commercial facility enabled us to design an experiment whereby we allocated two rearing treatments, one with ramps provided to access elevated structures and a control with elevated structures but no ramps and to alternate these treatments between the two rearing houses available to avoid treatment x house confounds. Each rearing flock was moved independently to a laying house with no mixing, so we were able to continue data collection and examine any long-term effects of the rearing treatment during the laying period. Rearing flocks were systematically allocated so that each laying house received one RR flock and one CR flock during the experiment.

Observations were made in the mornings at three time points during the rearing period at 1, 3 and 15–16 weeks, and three in the laying period at 16–17, 24 and 40 weeks of age. See Table 5 for a summary of experimental design, flock and housing information.

**Table 5:** Experimental design for each ramp reared and control reared flock for the 6 replicates. There were two rearing sheds used, Rear1 (R1) and Rear2 (R2), with 6 different laying sheds named A1, A2, B1, B2, C1 and C2.

Replicate	Rearing house	Laying house	Treatment at rear	Ramp angle at lay (degrees)
1	R1	B2	RR	30
	R2	B1	CR	30
2	R1	C2	CR	30
	R2	C1	RR	30
3	R1	A1	RR	45
	R2	A2	CR	45
4	R1	B2	CR	30
	R2	B1	RR	30
5	R1	C2	RR	30
	R2	C1	CR	30
6	R1	A1	CR	45
	R2	A2	RR	45

The rearing sheds were static with 142.7 m^2^ of floor space covered with wood shavings. Rearing sheds were both set up with feed tracks giving mini pellet feed up to 11 weeks of age then pellet grower feed and 7 nipple drinker lines. The lighting schedule was 23 h light in the first day reducing gradually over the rearing period to 10 h light at 7 weeks of age. A minimum light intensity of 10 lx is required, but with windows and pop-holes light intensity was higher in the houses. The temperature was maintained at 30 °C during the first few days then slowly reduced to match the temperature in the laying sheds. Shed heating was provided by gas spot lamps, whole shed heating through hot pipes running along the length of the shed and hot air fans run by a biomass boiler. All flocks had access to the outside range by 10 weeks of age through two pop holes (each L: 2 m by H: 0.4 m). Flocks were moved the short distance from the rearing to the laying house at between 15 to 16 weeks of age in one night using transport modules.

**Figure 3:**
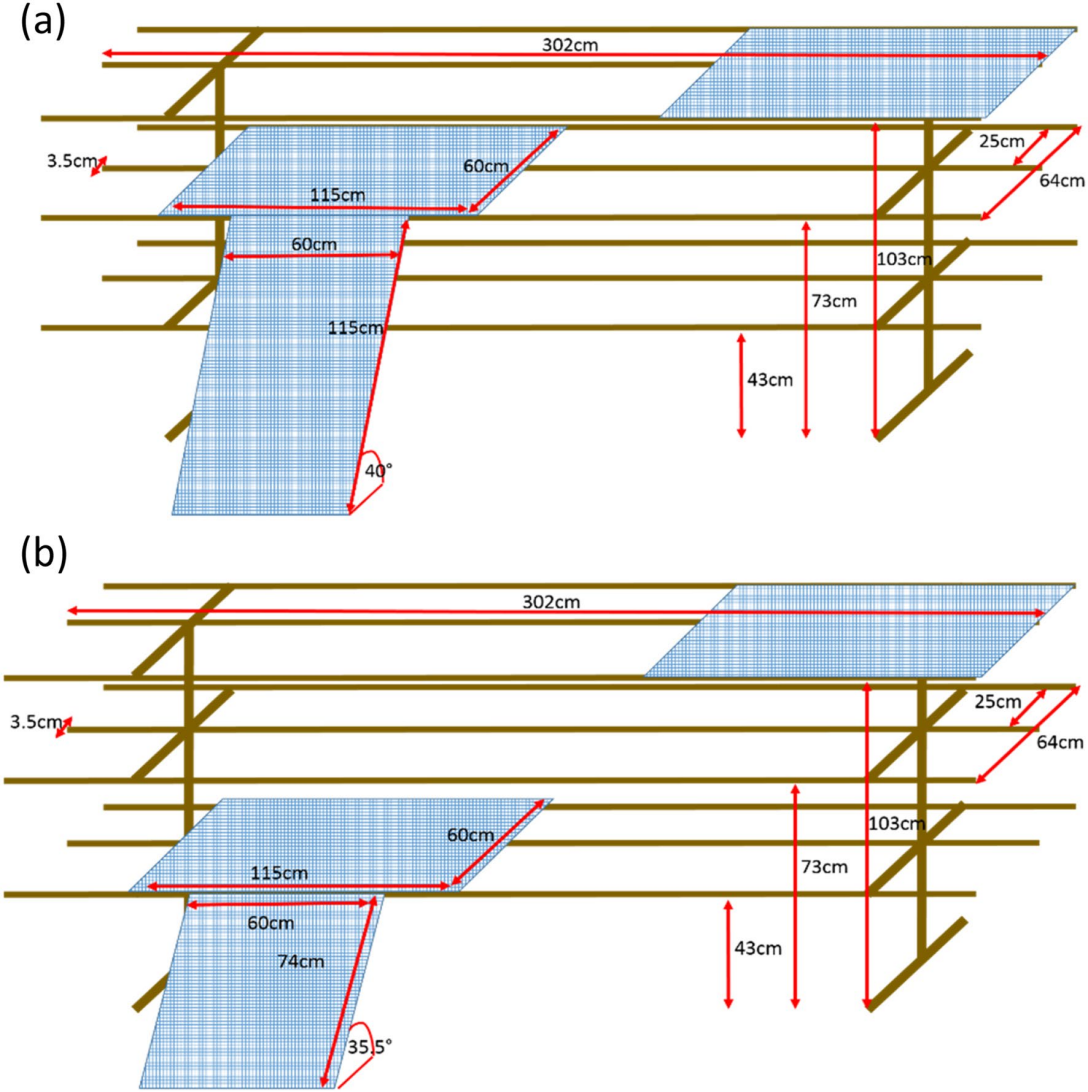
Elevated structure dimensions used in the ramp reared sheds, **(a)** shows the high ramp **(b)** shows the low ramp. The control sheds elevated structures were identical to these but without ramps.

All rearing flocks had access to six elevated structures (ES) (see Fig. 3) from four days of age when the chicks were released from the brooding circles. Each ES comprised nine metal perches (length 302 cm, width 3.5 cm), with three perches (25 cm apart) at three different heights (43 cm, 73 cm and 103 cm). Two plastic grids (width 60 cm, length 115 cm) were fixed within the ES to provide platforms at different heights (Fig. 3). In each replicate, the RR flock had one ramp attached to each ES. Three of the ES were fitted with plastic grid ramps (width 60 cm, length 74 cm, angle 35.5°) leading up to the low perch and three ES had ramps (width 60 cm, length 115 cm, angle 40°) leading up to the middle perch. The CR flock had six ES without ramps.

**Figure 4:**
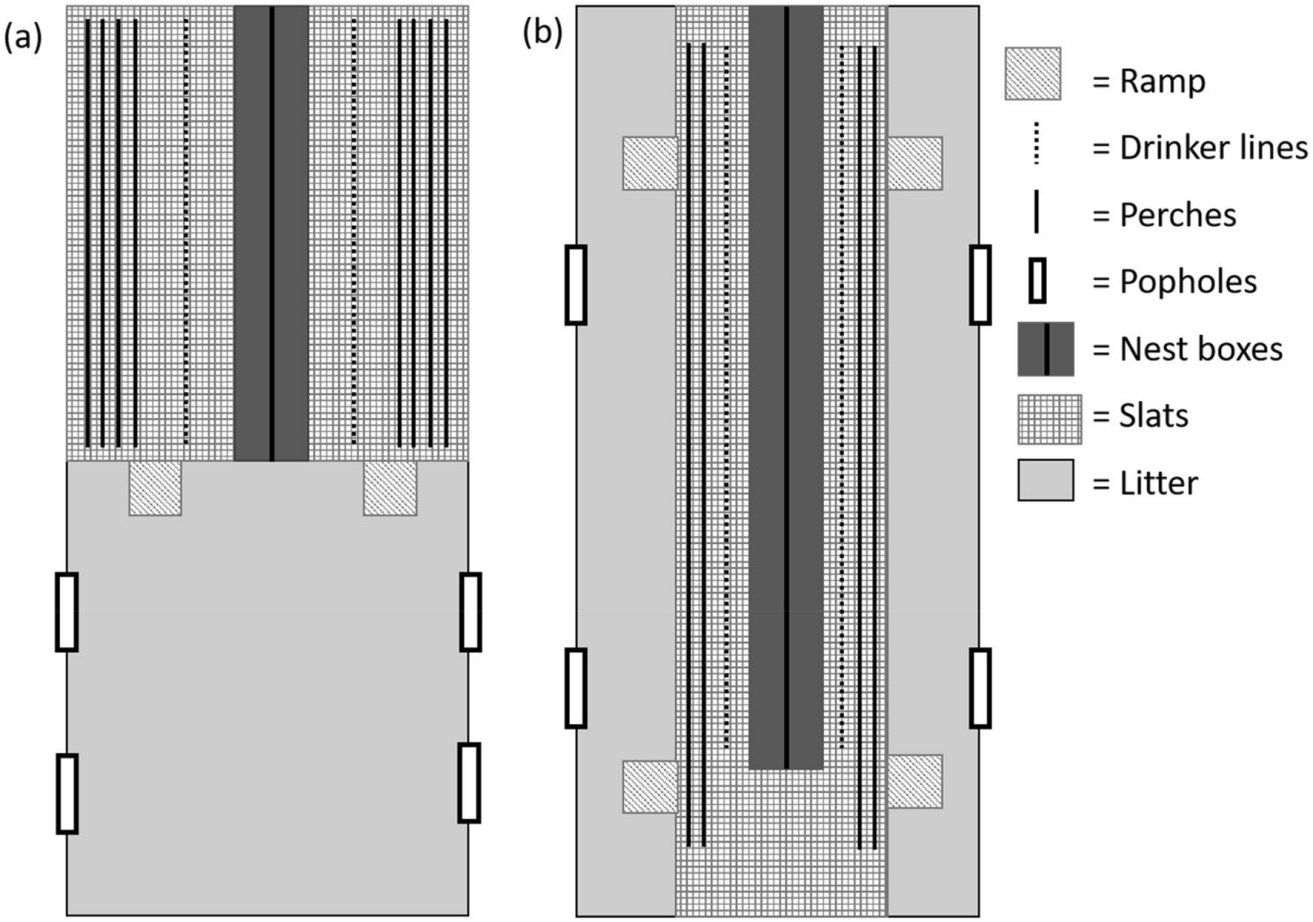
Plan view of the laying house layout **(a)** for replicates 1, 2, 4 and 5 and **(b)** for replicates 3 and 6. Images not to scale.

The six single-tier laying houses on-site were mobile organic units with approximately 345m^2^ of floor space. See Fig. 4 for a schematic plan of their layout. All had a raised area comprising plastic slats over supports (approx. 70 cm from the litter) and a ground-level litter area covered with wood chip. Four of the sheds (Fig. 4a) were set up with the slatted area spanning the whole width of the shed and halfway down the length. In two of the sheds (Fig. 4b) the litter area was either side of the elevated slatted area. Nest boxes ran down the centre of the slatted area, dividing this into two sections. Intermittent ramps were installed at the level change, resulting in 4 m of ramp access and 4 m without ramps in the shed with litter at the end and 8 m of ramp access and 13 m without ramps in the shed with litter at the sides. In sheds A1 and A2 the height of the slatted area resulted in a steeper ramp angle of 45° compared to 30° in the other sheds. There were four pop holes at ground level with two on each side of the house (L: 2.35 m by H: 0.4 m) leading to the range from the litter area on both sides of the sheds. All sheds had aerial perches at 1 m high with 18 cm of perching space per bird resulting in approximately 360 m of perch length running the length of the slatted area. Feed tracks and drinker lines matched those in the rearing sheds. The lighting schedule was 16 h of light and varied between summer and winter with the lights set to turn off at the same time as natural dusk. The birds were fed on organic mini pellets throughout lay. Enrichment was provided to the flocks in the form of pecking objects such as buckets and boots. Replicates 5 and 6 were provided with pecking blocks and alfalfa hay nets hung on the litter area.

### Assessments of behaviour

Observations were made at three time points during the rearing period at 1, 3 and 15–16 weeks. On the first visit at 1 week of age, the total number of chicks on each ES was counted once in spot counts in the morning. At 3 and 15–16 weeks, observations of the movements up and down the ES were made. Three of the 6 structures were chosen at random in each shed. The number of chicks present on the different parts of the ES was counted at the beginning and end of the recording period to allow a comparison with the 1-week counts. The recordings involved 5-min continuous sampling where all movements down the ES were recorded and the area the chicks moved down from was noted. This was then repeated for movements up the ES. Focal bird recordings were taken at 3 and 15–16 weeks of age. Records were made for each of 3 randomly selected ES. When 10 focal birds had been observed (approximately 30 birds per flock), or 10 min had passed recordings stopped. A focal bird was chosen if it was performing orientation behaviour, indicating a downwards or upwards transition. This was described as the bird rotating its head to look in the direction of movement. Behaviours performed after the orientation behaviour were tallied, thus recorded as counts per behaviour (see Table 6). Recordings were stopped if birds completed a transition or moved away from transitioning.

**Table 6:** An ethogram of behaviours of focal birds during up and down movements.

Type of transition	Behaviour	Description
Pre-transition behaviours	Orientation	Bird rotates head to look in the direction of movement
	Pacing	Bird raises one or both feet and replaces on ground or perch, or bird moves along structure followed by head orientation, crouch or movement down/up
	Crouching	Lowers body to the ground, or lowers body on ramp
Transition behaviours	Straight down	Bird walks or runs straight down the ramp
	Zig Zag down	Bird crossed ramp in a zig zag motion to get to the bottom or top
	Wing flap	Bird flaps wings during movement
Move away	Move away	The bird moves away from the initial position without showing further intention of traversing within 10 s
	To ramp	Bird moves away but to ramp

At 15–16 weeks of age, three types of interactions were recorded for feather pecking. These included severe feather pecking (SFP), gentle feather pecking (GFP) and aggressive pecks (AP)^28^. A quadrat area 2 m by 2 m was randomly selected, with the number of birds in each quadrat counted at the beginning and end of the recording period. The number of SFP, GFP and AP were recorded over three minutes of continuous recordings in three different areas of the house, selected randomly at each end and the middle of the shed. Feather pecks and aggressive pecks were recorded as bouts: a series of pecks not separated by more than 5 s^28^. Rates of pecking were calculated as the number of pecks per bird per second.

In the laying shed around 16–17 and at 24 weeks of age 3-min continuous sampling and focal bird recordings were taken for transitions between the slats and litter. Four recordings were made at 2-m lengths along the elevated slatted area: two areas with ramps (RA) and two areas without ramps (NRA) were selected. Separate recordings were taken for upwards and downwards movements and the number of birds in the recording area were counted at the start and end of the scans. At 16–17 and 24 weeks of age, feather pecking observations were taken using the same procedure as for the 15–16-week observations during rear.

### Welfare assessments and production data rearing phase

Feather scores of 20 birds per flock were recorded at 16–17 weeks of age by walking in a straight line down the centre of the shed, selecting a bird at random then counting two birds to the left of this and visually feather scoring that bird. Birds were not handled to minimise disturbance and plumage was scored using the method from Bright et al.^33^. The neck, back, rump, tail and wings were scored using a four-point scale 0 (best) to 4 (worst). Data were obtained from the farm records for percentage cumulative mortality and body weight.

### Welfare assessments and production data laying phase

At 16–17 and 24 weeks of age, the attitude of the flocks was assessed using the approach distance and reactions to novel objects methodology developed by Whay et al.^34^. Distance to approach birds before they moved away was recorded by walking through the house selecting a bird at random and counting two birds to the left. The bird had to be standing up and facing the researcher, who approached the bird at a steady pace and recorded the distance before the bird moved away. This was repeated on 20 birds in each flock. Reactions to a novel object (blue folder at 17 weeks of age and a white and blue tub at 24 weeks of age) were assessed by placing a novel object on the ground and recording the time taken for the first bird to interact with it and then how many birds were within a 30 cm radius after 60 s. The novel object test was repeated in 4 areas per flock. Range use was recorded by counting the number of birds near to the house (5 m) in the middle range (5–20 m) and far (the rest of the range). Feather scores of 20 birds per flock were recorded at 17 and 24 weeks of age using the same procedure as for the 16-week assessment for birds at rear.

At 40 weeks of age, feather cover and keel bone fractures were scored. Up to 100 birds per shed were caught from four different locations (25 litter, 25 slats, 25 perches, 25 nest boxes). In four sheds only 50 birds were caught as the birds were fearful and showed signs of distress. Feather cover was scored by picking the bird up and scoring the body and flight feathers separately using a the AssureWel three-point scale 0 (best) to 2 (worst)^35^. The keel damage was then scored using a 0 (no damage) to 2 scale based on the technique used by Wilkins et al.^36^. Validation for keel bone palpations was conducted. A score of 94% matched scores compared to an experienced gold standard assessor and 85% match at dissection for scoring a break. At 24 weeks of age, the number of floor eggs were counted over 1 day.

Data were collected from the farm records on laying house percentage of daily eggs, average egg weight (grams), average hen body weight and feed conversion ratio.

During the 16 week recordings in the final rearing flocks, the lighting inside the shed was considerably reduced compared to previous flocks. This resulted in poor visibility for feather cover and feather pecking observations, so these were not taken during this visit. Data were not obtained on keel fractures and feather cover scores at 40 weeks for the first laying flocks visited as their sheds were destroyed by strong winds.

### Statistical analysis

Data were analysed using SPSS 24 (IMB) or MLwiN 3.0. The statistical package MLwiN was chosen as it is designed for multilevel modelling and can therefore accommodate data nested within levels with repeated measures. Such models account for dependence between responses caused by grouping of birds within sheds, and repeated measures taken from the same sheds on different visits within and between replicates. Including visit and replicate as nested effects ensures that dependences (e.g. due to differing times of year when data were collected) are accounted for. All residuals were checked for normal distributions using a Shapiro-Wilks test or plotted graphically and no transformations were needed to meet the assumptions of the tests. All results are reported in the format mean ± SD unless when stated as the percentage of birds performing a behaviour during transitions.

### Assessments of behaviour

At rear, from the counts of chicks on structures and counts of transitions up and down the structures, a normal model (generalised linear model) was used with a four-level hierarchy (bird within shed within visit within replicate). The same normal model and four level hierarchy were used for the counts of transitions in the laying shed.

For the focal bird behaviours of birds transitioning at rear and lay, the data were presented as the percentage of each behaviour calculated for the birds in the recording session for the two rearing treatments. The direction (up or down) was analysed separately. For the focal birds at lay, all were included in the analysis for the pre-transitioning behaviours, only birds that attempted a transition were included for analysis of the transitioning behaviours. Pre-transition behaviours for birds that moved-away and did not transition were analysed separately. Owing to the low occurrence of behaviours during the focal recordings for transitions up and down the ramps, data were coded as yes or no, and a Binomial model was used for analysis for both the rear and lay focal transition data with four hierarchical levels (bird within shed within visit within replicate).

### Welfare and production data

For the Novel object test, human approach, feather pecking and feather cover data a normal model was used in MLwiN with four-levels (Bird within Shed within visit within replicate). Floor eggs were analysed using a two-tailed t-test in SPSS, due to limited data. Ordinal data such a keel bone fracture scores and feather cover recorded at 40 weeks of age were converted to binomial data due to a lack of data for some scores, these were therefore analysed using a binomial model in MLwiN with two levels (Bird within shed).

Production data at rear (body weight in grams) and lay (% eggs daily, egg weight in grams, body weight in grams and feed conversion ratio) were obtained from farm records and analysed in SPSS using a general linear model with treatment (CR and RR) as a fixed factor and age (3, 8 and 14 weeks at rear and 20, 30 and 70 weeks at lay) as a random factor to account for repeated results. Cumulative percentage mortality was analysed at 14 weeks of age using a t-test to compare the treatment groups.

### Ethical approval

Ethical approval for this project was granted by the University of Bristol’s Animal Welfare and Ethical review body under UIN: UB/16/040 and all methods were conducted in accordance with the review body and UK legislation.
